# Perioperative Anesthesia Strategies for the Prevention of Postoperative Nausea and Vomiting Within Enhanced Recovery After Surgery Pathways: A Clinical Narrative Review

**DOI:** 10.3390/jcm15103829

**Published:** 2026-05-15

**Authors:** Rachel Dombrower, Alyssa McKenzie, Andrew J. Tucker, Johnathan Atwell

**Affiliations:** 1 School of Medicine, St. George’s University, West Indies 11739, Grenada; 2 Department of Anesthesiology, University of Miami Miller School of Medicine, Miami, FL 33146, USA; atucker@chiefanesthesia.com; 3 Department of Biological Sciences, Nova Southeastern University, Fort Lauderdale, FL 33328, USA

**Keywords:** postoperative nausea and vomiting (PONV), Enhanced Recovery After Surgery (ERAS), perioperative anesthesia, antiemetic prophylaxis, multimodal therapy, risk stratification, total intravenous anesthesia (TIVA), opioid-sparing anesthesia

## Abstract

Postoperative nausea and vomiting (PONV) remain a leading preventable perioperative complication despite advances in anesthetic and surgical care, significantly affecting recovery within Enhanced Recovery After Surgery (ERAS) pathways. ERAS protocols provide a structured, multidisciplinary framework for perioperative optimization; however, variability in the implementation of PONV prevention strategies persists. This narrative review synthesizes current evidence on perioperative strategies for PONV prevention within ERAS pathways, focusing on patient risk stratification, multimodal pharmacologic prophylaxis, anesthetic techniques, and adjunctive non-pharmacologic interventions. We evaluate validated risk prediction tools, including the Apfel score, and highlight the importance of individualized prophylactic strategies based on patient, surgical, and anesthetic risk factors. Multimodal antiemetic regimens, opioid-sparing anesthesia, total intravenous anesthesia (TIVA), and regional techniques are discussed as key components of perioperative management. In addition, non-pharmacologic interventions such as optimized fluid therapy, early mobilization, and supportive perioperative care are reviewed as integral elements of ERAS-based recovery pathways. Complementing existing consensus guidelines, this review provides a practical, workflow-based framework spanning preoperative risk assessment, intraoperative decision-making, and postoperative monitoring for direct application within ERAS protocols.

## 1. Introduction

Postoperative nausea and vomiting (PONV) remain among the most common and distressing complications following surgery, affecting approximately 20–30% of the general surgical population and up to 80% of high-risk patients [1,2,3]. Beyond patient discomfort, PONV is associated with delayed recovery, prolonged hospital length of stay, and complications including dehydration, electrolyte imbalance, wound dehiscence, and aspiration [4]. Despite advances in anesthetic and perioperative care, its prevention remains challenging due to a complex, multifactorial etiology involving patient-related, surgical, and anesthetic factors [3,5].

Enhanced Recovery After Surgery (ERAS) protocols represent a multidisciplinary, evidence-based approach to perioperative care that aims to improve clinical outcomes, accelerate recovery, and shorten hospital stays [6]. These pathways integrate strategies across the preoperative, intraoperative, and postoperative periods, including opioid-sparing analgesia, early mobilization, and optimized nutritional support. Within this framework, effective prevention of PONV is essential, as poorly controlled symptoms can hinder recovery, delay discharge, and negatively impact patient satisfaction [7].

Although numerous pharmacologic and non-pharmacologic interventions for PONV have been described, variability in implementation and integration within ERAS pathways persists. This review synthesizes contemporary evidence on perioperative strategies for PONV prevention within ERAS pathways, emphasizing practical clinical application across the preoperative, intraoperative, and postoperative phases of ERAS-based care. While existing consensus guidelines, including the Fourth Consensus Guidelines for the Management of PONV (Gan et al., 2020 [2]), provide evidence-based pharmacologic recommendations, their focus remains primarily on antiemetic selection rather than the practical embedding of prevention strategies within perioperative workflows. The present review complements existing literature by adopting a practical, workflow-based focus. Specifically, it provides a phase-specific framework spanning preoperative risk assessment, intraoperative decision-making, and postoperative monitoring that perioperative teams can apply directly within ERAS protocols.

## 2. Methods

This narrative review examines perioperative strategies for the prevention of postoperative nausea and vomiting (PONV) within Enhanced Recovery After Surgery (ERAS) pathways. Although a formal systematic review methodology was not employed, the literature search and selection process followed a structured and transparent approach to ensure comprehensive and balanced representation of the available evidence. For the literature search, relevant publications were identified through structured searches of three electronic databases: PubMed, Embase, and Google Scholar. Searches were conducted using combinations of the following terms: “postoperative nausea and vomiting,” “PONV,” “Enhanced Recovery After Surgery,” “ERAS,” “antiemetic prophylaxis,” “anesthesia,” “total intravenous anesthesia,” “TIVA,” “opioid-sparing anesthesia,” “risk stratification,” “multimodal analgesia,” and “perioperative outcomes.” Database searches were supplemented by manual review of reference lists from key articles, consensus statements, and clinical practice guidelines to identify additional relevant sources not captured in the primary search. The search primarily focused on English-language studies published within the past 15–20 years (approximately 2005–2026), reflecting the period during which ERAS protocols became widely adopted and PONV consensus guidelines were established and updated. Earlier trials and foundational consensus statements were included where appropriate given their continued clinical relevance. All included studies were published in English. Evidence published through January 2026 was considered. The inclusion and exclusion criteria included studies based on their direct relevance to perioperative PONV prevention within ERAS pathways. Priority was given to randomized controlled trials (RCTs), meta-analyses, systematic reviews, and major clinical practice guidelines. High-quality observational studies and large-scale perioperative cohort studies were also incorporated where RCT-level evidence was limited. Case reports, editorials, and studies not directly applicable to perioperative or ERAS contexts were excluded. Within the evidence appraisal and synthesis, while formal quality appraisal tools were not applied given the narrative methodology, the strength and source of supporting evidence were considered throughout the review. Priority was given to peer-reviewed publications in high-impact journals, studies with large sample sizes, and sources cited within major clinical practice guidelines. Evidence statements throughout the manuscript are qualified according to study design, with meta-analyses and consensus guidelines given the highest weight, followed by RCTs, and then observational or expert consensus-based evidence where higher-quality data are lacking. Selected literature was organized thematically according to patient risk assessment, surgical and anesthetic risk factors, validated risk stratification tools, pharmacologic prophylaxis strategies, anesthetic considerations, non-pharmacologic interventions, and integration within ERAS frameworks.

## 3. Risk Assessment for PONV

Accurate preoperative risk assessment is the foundation of effective PONV prevention within ERAS pathways, enabling clinicians to tailor prophylactic strategies to individual patient need. Risk factors are broadly categorized as patient-specific, surgical, and anesthetic in nature, each contributing independently and additively to overall PONV risk.

### 3.1. Patient-Specific Risk Factors

Patient characteristics are among the strongest predictors of postoperative nausea and vomiting (PONV) [3]. Female sex, younger age, non-smoking status, and a personal or family history of motion sickness or prior PONV are consistently associated with increased risk [3]. These factors form the basis of widely used predictive models and exhibit an additive effect, with risk increasing substantially as multiple factors coexist. Accordingly, individualized risk assessment is essential, as patients with higher baseline risk derive the greatest benefit from multimodal prophylactic strategies [2].

### 3.2. Surgical and Anesthetic Risk Factors

Surgical and anesthetic factors further contribute to PONV risk. Procedures such as laparoscopic, gynecologic, breast, and otolaryngologic surgeries are associated with higher incidence, likely due to pneumoperitoneum, visceral manipulation, and increased postoperative pain [8]. Prolonged surgical duration is an independent risk factor. Anesthetic technique plays a central role, with volatile anesthetics and nitrous oxide increasing risk, while total intravenous anesthesia (TIVA) with propofol is associated with reduced incidence [9,10]. Perioperative opioid use remains a major modifiable contributor, highlighting the importance of opioid-sparing and multimodal analgesic strategies within ERAS protocols [11,12,13]. Collectively, optimization of anesthetic management represents a key opportunity for targeted PONV risk reduction within perioperative care pathways.

### 3.3. Risk Stratification Tools

Validated risk stratification tools provide a practical framework for guiding PONV prophylaxis [14,15]. The Apfel simplified risk score remains the most widely used model, assigning one point each for female sex, history of PONV or motion sickness, non-smoking status, and anticipated postoperative opioid use. PONV risk increases in a stepwise manner from approximately 10% with no risk factors to up to 80% in patients with all four [14]. Patients are stratified into low (0–1), moderate (2), and high (≥3) risk categories, which guide prophylactic antiemetic intensity [16,17]. Current consensus supports multimodal prophylaxis, with at least two antiemetic agents recommended in most patients and escalation in high-risk individuals [17,18]. Integration of risk stratification into ERAS pathways facilitates standardized perioperative decision-making and supports timely escalation of preventive strategies [16,17]. In real-world ERAS implementation, Apfel scoring is ideally performed during the preoperative anesthesia assessment, allowing results to be embedded directly into standardized order sets and perioperative checklists. Ensuring that antiemetic selection, anesthetic technique, and analgesic planning are coordinated prospectively rather than reactively helps reduce reliance on individual provider judgment while minimizing care variability across multidisciplinary teams [16,19]. Key patient-specific, surgical, and anesthetic risk factors, along with their modifiability, are summarized in Table 1.

## 4. Pharmacologic Strategies

Pharmacologic prophylaxis forms the cornerstone of PONV prevention within ERAS pathways, particularly in patients with moderate or high baseline risk [2,20]. Contemporary strategies emphasize multimodal therapy targeting complementary emetogenic pathways to enhance efficacy compared with single-agent prophylaxis.

### 4.1. Common Antiemetic Agents

5-HT_3_ receptor antagonists, such as ondansetron, are widely used as first-line agents due to their proven efficacy and favorable safety profile [20,21]. Corticosteroids, particularly dexamethasone administered at induction of anesthesia, provide additive antiemetic effects and enhance the efficacy of combination regimens [22,23]. Neurokinin-1 (NK1) receptor antagonists, such as aprepitant, offer additional benefit in high-risk or highly emetogenic surgical settings, including laparoscopic and prolonged procedures [2,24,25,26]. Dopamine antagonists (e.g., droperidol, metoclopramide) and antihistamines may be used as adjuncts or for rescue therapy within multimodal prophylactic or treatment protocols [2].

Despite their widespread use, important limitations exist within this drug class. While ondansetron remains the most studied 5-HT_3_ antagonist, comparative evidence suggests that palonosetron may offer superior efficacy due to its longer half-life and allosteric receptor binding properties, though its higher cost limits routine use [20,21]. Additionally, pharmacogenomic variability in CYP2D6 metabolism affects ondansetron efficacy in a clinically meaningful proportion of patients, representing an area of ongoing controversy and a rationale for individualized antiemetic selection [27].

### 4.2. Multimodal Antiemetic Regimens

Evidence strongly supports combination therapy for patients at moderate or high risk of PONV, with current practice favoring regimens that target complementary emetogenic pathways [2,20]. The most common approach combines a 5-HT_3_ receptor antagonist with dexamethasone, then escalating to include an NK1 antagonist or dopamine antagonist in higher-risk patients [16,22,28]. Timing of administration is important: corticosteroids are most effective at induction, whereas 5-HT_3_ antagonists are generally given near the end of surgery [29,30]. When appropriately implemented, multimodal regimens reduce PONV incidence, decrease rescue antiemetic use, improve patient satisfaction, and have demonstrated cost-effectiveness by reducing unplanned admissions and delays in discharge, particularly in ambulatory settings [22,31]. However, important limitations in the existing evidence base warrant acknowledgment. Many RCTs are limited by heterogeneous patient populations, variable surgical contexts, inconsistent outcome definitions, and short follow-up periods that do not capture late PONV beyond 24 h. The optimal number, combination, and sequencing of agents for specific surgical populations remains incompletely defined, and current guideline recommendations are largely extrapolated from general surgical cohorts rather than procedure-specific trials [2,18].

### 4.3. Tailoring Therapy to Risk

PONV prophylaxis should be tailored according to individual risk profiles to optimize efficacy while minimizing unnecessary medication exposure. Low-risk patients may not require routine prophylaxis or may receive a single antiemetic agent, whereas moderate-risk patients benefit from dual-agent therapy combining agents with different mechanisms of action [16,21]. High-risk patients require intensified multimodal prophylaxis, typically involving three or more antiemetic classes, alongside optimization of anesthetic technique and incorporation of non-pharmacologic measures [2,16]. This risk-adapted approach allows targeted escalation of therapy based on patient and procedure-related factors, including prior history of PONV and anticipated opioid requirement. Effective perioperative management relies on integrating pharmacologic prophylaxis with individualized anesthetic planning, ensuring that preventive strategies are appropriately matched to patient risk while concurrently avoiding overtreatment in low-risk populations. Risk-stratified pharmacologic prophylaxis strategies are summarized in Table 2. Within ERAS pathways, this individualized risk assessment and prophylactic planning ideally occurs during the preoperative anesthesia consultation, allowing antiemetic selection, anesthetic technique, and analgesic strategy to be coordinated as a unified perioperative plan prior to the day of surgery.

## 5. Anesthetic Considerations

Anesthetic management plays a pivotal role in the perioperative strategies for preventing PONV and complements pharmacologic prophylaxis within ERAS pathways [16,32]. Careful selection of anesthetic technique and intraoperative adjuncts can reduce emetogenic stimuli and directly influence overall PONV risk [16,33].

### 5.1. Choice of Anesthetic Technique

The type of anesthesia administered influences PONV risk. Volatile inhalational agents, including sevoflurane and desflurane, are associated with higher PONV risk, whereas TIVA using propofol exerts an intrinsic antiemetic effect and is preferred in higher-risk patients [2,16]. Nitrous oxide, commonly used as an adjunct, has also been associated with increased PONV and is generally avoided or minimized in susceptible individuals [34,35]. Regional anesthesia techniques, including neuraxial and peripheral nerve blocks, are associated with reduced PONV incidence and are an important component of anesthetic planning [16,36]. In appropriate surgical settings, the combination of regional anesthesia with TIVA offers synergistic benefits by minimizing both inhalational anesthetic exposure and opioid consumption [16,37]. Despite its established antiemetic benefits, TIVA’s impact on longer-term outcomes and mortality compared with inhalational techniques remains an area of ongoing investigation, highlighting the importance of individualized anesthetic selection beyond PONV prevention alone [9,10]. Additionally, practical implementation of TIVA requires familiarity with target-controlled infusion systems, which may limit adoption in lower-resource settings.

### 5.2. Opioid-Sparing Strategies

Perioperative opioid use is a well-established, dose-dependent risk factor for PONV [13,16]. As such, opioid-sparing approaches are a key component of perioperative pain management within ERAS pathways. Non-opioid analgesic regimens incorporating agents such as acetaminophen, NSAIDs, and gabapentinoids, with or without regional techniques, provide effective pain control while reducing opioid exposure and associated emetogenic risk [36,38,39]. Regional techniques further decrease systemic opioid requirements and enhance postoperative recovery. In high-risk patients, limiting opioid use should be integrated with anesthetic planning and antiemetic prophylaxis to optimize perioperative outcomes [2,16,17]. Within ERAS pathways, opioid-sparing strategies are ideally identified and planned during preoperative assessment, implemented intraoperatively through multimodal analgesic regimens and regional techniques, and continued postoperatively to minimize opioid-related emetic burden during recovery.

### 5.3. Adjunctive Measures

Additional intraoperative strategies may further reduce PONV. Avoidance of large fluid shifts and intraoperative hypotension along with maintenance of normothermia have been associated with reduced PONV incidence [40,41,42]. Depth of anesthesia monitoring may help limit excessive anesthetic exposure and reduce the emetogenic effects of volatile agents [43,44]. Perioperative patient education and expectation management may also influence postoperative symptom reporting and overall patient experience, supported by Cochrane review evidence [45], randomized trial data [46], and observational findings [47], though effect sizes remain modest. Collectively, these measures provide added opportunities to reduce PONV through optimization of intraoperative physiology and perioperative care.

## 6. Non-Pharmacologic and Adjunctive Approaches

Although pharmacologic prophylaxis and anesthetic optimization form the foundation of PONV prevention, non-pharmacologic strategies serve as important adjuncts within ERAS protocols [2,16]. These strategies enhance antiemetic efficacy, reinforce multimodal prophylaxis, and support patient-centered recovery.

### 6.1. Perioperative Hydration and Fluid Management

Optimizing perioperative fluid status is central to PONV reduction. Hypovolemia and dehydration increase emetic symptoms, likely through impaired tissue perfusion and gut ischemia [48]. ERAS protocols recommend goal-directed fluid therapy to maintain euvolemia [16]. Adequate intravenous hydration, guided by hemodynamic monitoring and individualized patient factors, has been shown to reduce postoperative nausea incidence and severity, supported by moderate-quality evidence from a Cochrane systematic review of supplemental perioperative intravenous crystalloids [41].

### 6.2. Early Oral Intake and Mobilization

Early oral intake and ambulation, which are hallmarks of ERAS protocols, contribute directly to PONV prevention [16]. Once patients are clinically stable, resuming light oral fluids as soon as possible supports gastrointestinal motility and may reduce nausea, while early mobilization promotes gut function and reduces ileus risk. Although direct RCT-level evidence specifically linking early oral intake and ambulation to reduced PONV incidence remains limited, these interventions are consistently recommended within ERAS consensus guidelines based on their broader recovery benefits and mechanistic plausibility [16]. Effective analgesic management should accompany these interventions to support patient comfort and safety.

### 6.3. Complementary and Supportive Measures

Acupressure or acupuncture at the P6 (Neiguan) point has demonstrated modest but consistent antiemetic effects, supported by a Cochrane network meta-analysis of PC6 stimulation trials for PONV prevention [49]. Minimizing preoperative fasting follows current ASA guideline recommendations [50], and patient education regarding postoperative expectations has been associated with reduced PONV symptom burden based on moderate-quality randomized trial evidence [46]. Avoiding gut-distending medications and limiting noise, odors, and unnecessary movement in the recovery area are recommended on the basis of physiologic rationale and expert consensus, as robust RCT-level evidence remains limited, though trials are ongoing [51].

### 6.4. Integration into Multimodal Care

Effective PONV prevention requires integration of non-pharmacologic strategies with pharmacologic prophylaxis and anesthetic planning. While non-pharmacologic interventions alone are insufficient to replace multimodal pharmacologic prophylaxis in moderate- and high-risk patients, evidence supports their additive contribution when systematically incorporated into ERAS protocols [16]. Goal-directed fluid therapy and opioid-sparing analgesia represent the non-pharmacologic interventions with the strongest and most consistent evidence base for PONV reduction, while acupressure, patient education, and environmental measures provide clinically meaningful but more modest incremental benefits [41,46,49]. Within ERAS pathways, these interventions are incorporated into standardized perioperative protocols to ensure consistent, multimodal prevention, improving patient outcomes and satisfaction beyond what pharmacologic prophylaxis alone achieves [16].

## 7. Integration into ERAS Pathways

Effective PONV prevention requires coordinated, interdisciplinary collaboration across surgical, anesthesia, and nursing teams [19]. ERAS pathways facilitate this by embedding evidence-based strategies into standardized perioperative protocols, ensuring consistent application over isolated provider-dependent practice.

### 7.1. Standardized Protocols and Checklists

When embedded within ERAS protocols, PONV prevention becomes a structured, systematic process operationalized across three distinct perioperative phases [2,16]. In the preoperative phase, risk stratification using a validated tool such as the Apfel score guides antiemetic selection, informs anesthetic planning, establishes fasting minimization targets, and provides an opportunity for patient education. In the intraoperative phase, standardized order sets and checklists ensure timely implementation of key decisions, including anesthetic technique selection—favoring TIVA with propofol in moderate- and high-risk patients—avoidance of nitrous oxide, opioid-sparing multimodal analgesia, and goal-directed fluid therapy [16,19]. In the postoperative phase, nursing and clinical staff monitor for breakthrough PONV, implement non-pharmacologic supportive measures, administer rescue antiemetic therapy using a mechanism-switch approach when prophylaxis fails, and facilitate early oral intake and ambulation [2,16]. This phased framework minimizes care variability, reduces the likelihood of oversight, and ensures PONV prevention is applied as a continuously protocol-driven component of ERAS-based care rather than an isolated intervention.

### 7.2. Multidisciplinary Collaboration

ERAS emphasizes the importance of collaboration among anesthesiologists, surgeons, nurses, pharmacists, and other perioperative staff [16,19]. Shared awareness of each patient’s risk profile, anesthetic plan, and prophylactic strategy ensures that all team members can contribute meaningfully to prevention efforts. Nursing staff play a critical role in monitoring patients for early emetic symptoms, implementing non-pharmacologic interventions, and providing patient education throughout the recovery process.

### 7.3. Continuous Audit and Quality Improvement

**Figure 1:**
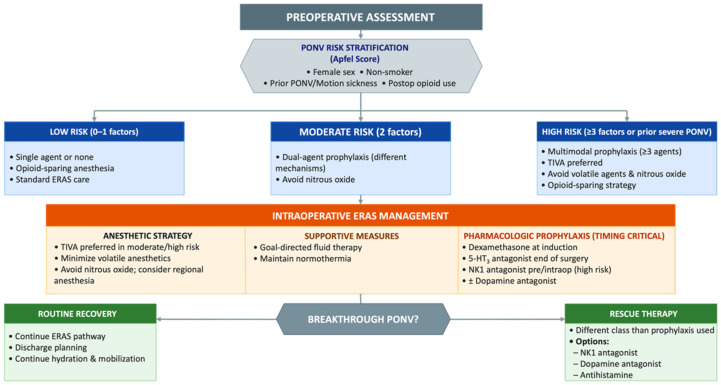
ERAS-based algorithm for the prevention and management of postoperative nausea and vomiting (PONV).

Integration of PONV prevention within ERAS pathways supports ongoing quality improvement through systematic outcome monitoring [52]. Recommended audit indicators include: PONV incidence at 24 h postoperatively; proportion of moderate- and high-risk patients receiving guideline-concordant multimodal prophylaxis; rescue antiemetic utilization in the post-anesthesia care unit and ward; time to first oral intake and ambulation; and patient-reported nausea severity at discharge [53,54]. Regular review of these indicators enables teams to identify gaps, refine protocols, and benchmark performance across providers and institutions [55,56]. This continuous improvement cycle integrates pharmacologic, anesthetic, and non-pharmacologic interventions into a cohesive management strategy, helping reduce PONV incidence while promoting better recovery and patient well-being [16,57,58]. A structured, risk-stratified perioperative approach integrating these elements is illustrated in Figure 1.

## 8. Future Directions

Despite advances in pharmacologic and non-pharmacologic prophylaxis, PONV continues to be a clinically significant challenge, notably in high-risk populations. Newer strategies may improve PONV prevention by more precise individual risk assessment and the incorporation of newer antiemetic agents into pharmacologic regimens. Ongoing research is focused on refining targeted approaches and optimizing their clinical application.

### 8.1. Personalized and Risk-Adapted Prophylaxis

Rather than using the same approach for each patient, emerging evidence in PONV prevention supports tailoring care towards each individual’s characteristics. By integrating patient-specific characteristics, risk factors, pharmacogenomic data, and procedure-related emetogenic risk, clinicians are better able to predict optimal antiemetic regimens for each patient [59,60]. This precision-based approach can improve prophylactic efficacy and minimize unnecessary pharmacologic exposure [27].

### 8.2. Novel Pharmacologic Agents and Combinations

Emerging antiemetic agents offer promising additions to the perioperative armamentarium for patients who are refractory to standard treatments or at highest emetogenic risk. NK1 receptor antagonists, including aprepitant and fosaprepitant, have demonstrated efficacy specifically in PONV prevention in randomized controlled trials and meta-analyses, particularly in high-risk and highly emetogenic surgical settings [24,25,26,28]. Olanzapine, a multi-receptor antagonist targeting dopaminergic, serotonergic, and histaminergic pathways, has shown early promise as a perioperative antiemetic agent, with preliminary randomized trial evidence supporting a role in both prophylaxis and rescue therapy in surgical patients [61,62]. Furthermore, current studies are investigating optimized timing, dosing, and sequencing of existing antiemetic combinations to strengthen clinical guidelines and improve PONV control across diverse surgical populations [2,20]. By acting on multiple emetic pathways, these strategies may enhance prophylactic effectiveness and decrease the need for rescue antiemetics.

### 8.3. Integration with ERAS Innovations

Future ERAS protocols are increasingly incorporating more advanced patient monitoring, such as hydration status, opioid consumption, and patient-reported symptoms to optimize PONV prevention [63,64,65]. Tools such as mobile health applications and electronic health record-based alerts can help clinicians individualize care and ensure timely administration of preventive interventions, reducing the risk of missed or delayed steps in PONV management [65,66,67]. These innovations further reduce variability and ensure consistency in care across patients and providers, thereby improving the reliability and quality of outcomes. Emerging approaches such as machine learning-based risk prediction models and real-time electronic health record-integrated decision support tools may further enhance individualized PONV prophylaxis.

### 8.4. Research Gaps and Opportunities

Significant gaps remain in the literature surrounding PONV prevention. Large-scale studies directly comparing individualized versus standardized prophylactic approaches are limited, as well as data on long-term outcomes, patient satisfaction, and cost-effectiveness [58,68,69]. Addressing these gaps are essential to support the sustainable integration of emerging strategies into ERAS pathways and advance patient-specific, evidence-driven care. Future research focusing on personalized prophylactic approaches and novel pharmacologic agents may improve PONV prevention. The integration of digital health technologies may also enhance recovery within ERAS pathways.

### 8.5. Limitations

Several limitations of the present review should be acknowledged. As a narrative review, the literature search and selection process followed a narrative rather than systematic methodology, and selection bias cannot be fully excluded. The evidence base draws predominantly from colorectal and general surgical populations, which may limit applicability to other specialties such as cardiac, thoracic, and pediatric surgery. Variability in ERAS protocol design and institutional resources across studies also limits direct comparability. Many included RCTs further rely on short follow-up periods and inconsistent outcome definitions, with late PONV beyond 24 h often underreported. While these limitations exist, the present review synthesizes the best available evidence to provide a clinically actionable framework for PONV prevention within contemporary ERAS pathways.

## 9. Conclusions

Effective PONV prevention within ERAS pathways requires a coordinated approach that encompasses individualized risk stratification, pharmacologic prophylaxis, anesthetic optimization, and non-pharmacologic interventions. To date, there is no single strategy that is superior in isolation; therefore, best outcomes are achieved when evidence-based measures are systematically integrated across interdisciplinary perioperative teams. Advances in pharmacogenomics, novel antiemetic agents, and digital health technologies offer promising opportunities to continue individualizing and refining prophylactic strategies. Continued research addressing existing gaps in long-term outcomes and cost-effectiveness will be important to sustain and further advance these improvements within evolving ERAS frameworks.

## Figures and Tables

**Table 1:** Risk Factors for Postoperative Nausea and Vomiting and Their Modifiability.

Risk Factor	Category	Modifiable	Clinical Implication
Female sex	Patient-specific	No	Higher baseline PONV risk; requires risk-adjusted prophylaxis
Younger age	Patient-specific	No	Higher baseline PONV risk
History of PONV or motion sickness	Patient-specific	No	Strong predictor; prioritize multimodal prophylaxis
Non-smoking status	Patient-specific	No	Higher baseline PONV risk
Use of volatile anesthetics	Anesthetic	Yes	Increased PONV risk; consider TIVA
Nitrous oxide use	Anesthetic	Yes	Avoid in high-risk patients
Perioperative opioid use	Perioperative	Yes	Minimize via opioid-sparing or multimodal analgesia
Type of surgery (laparoscopic, gynecologic, ENT, breast)	Surgical	Partially	Recognize high-risk procedures; increase prophylaxis accordingly
Duration of surgery	Surgical	Partially	Longer duration increases risk; adjust prophylaxis as needed

**Table 2:** Risk-stratified multimodal pharmacologic prophylaxis for PONV within ERAS pathways.

PONV Risk Level	ERAS Phase	Recommended Prophylaxis Strategy	Pharmacologic Regimen (Examples)	Timing Considerations	Key ERAS Considerations
**Low (0–1 risk factors)**	Preoperative assessment Intraoperative	Minimal or single-agent prophylaxis	5-HT_3_ antagonist OR dexamethasone	Dexamethasone at induction; 5-HT_3_ antagonist at end of surgery if used	Opioid-sparing anesthesia; prophylaxis may be omitted in very low-risk cases
**Moderate (2 risk factors)**	Preoperative assessment Intraoperative	Dual therapy (two agents with different mechanisms)	5-HT_3_ antagonist + dexamethasone ± dopamine antagonist	Dexamethasone at induction; 5-HT_3_ antagonist near end of surgery	Avoid nitrous oxide; consider TIVA in selected patients
**High (≥3 risk factors or prior severe PONV)**	Preoperative assessment Intraoperative Postoperative monitoring	Multimodal prophylaxis (3–4 agents)	5-HT_3_ antagonist + dexamethasone + NK1 antagonist ± dopamine antagonist	Dexamethasone at induction; 5-HT_3_ at emergence; NK1 antagonist pre-op	TIVA preferred; opioid-sparing strategy; avoid nitrous oxide; consider regional anesthesia
**Rescue (any risk level with breakthrough PONV)**	Postoperative	Mechanism-switch therapy	Antiemetic from different class than prophylaxis used	Administer after symptom onset	Escalate therapy; reassess contributing anesthetic factors

## Data Availability

No new data were created or analyzed in this study.
